# Sprouty2 in the Dorsal Hippocampus Regulates Neurogenesis and Stress Responsiveness in Rats

**DOI:** 10.1371/journal.pone.0120693

**Published:** 2015-03-30

**Authors:** Antonia L. Dow, Tiffany V. Lin, Elena H. Chartoff, David Potter, Donna L. McPhie, Ashlee V. Van’t Veer, Allison T. Knoll, Kristen N. Lee, Rachael L. Neve, Tarun B. Patel, Dost Ongur, Bruce M. Cohen, William A. Carlezon

**Affiliations:** 1 Department of Psychiatry, Harvard Medical School, McLean Hospital, Belmont, Massachusetts, United States of America; 2 Viral Gene Transfer Core Facility, Massachusetts Institute of Technology, Cambridge, Massachusetts, United States of America; 3 Department of Pharmacology, Loyola University Chicago, Stritch School of Medicine, Maywood, Illinois, United States of America; Max Planck Institute of Psychiatry, GERMANY

## Abstract

Both the development and relief of stress-related psychiatric conditions such as major depression (MD) and post-traumatic stress disorder (PTSD) have been linked to neuroplastic changes in the brain. One such change involves the birth of new neurons (neurogenesis), which occurs throughout adulthood within discrete areas of the mammalian brain, including the dorsal hippocampus (HIP). Stress can trigger MD and PTSD in humans, and there is considerable evidence that it can decrease HIP neurogenesis in laboratory animals. In contrast, antidepressant treatments increase HIP neurogenesis, and their efficacy is eliminated by ablation of this process. These findings have led to the working hypothesis that HIP neurogenesis serves as a biomarker of neuroplasticity and stress resistance. Here we report that local alterations in the expression of Sprouty2 (SPRY2), an intracellular inhibitor of growth factor function, produces profound effects on both HIP neurogenesis and behaviors that reflect sensitivity to stressors. Viral vector-mediated disruption of endogenous Sprouty2 function (via a dominant negative construct) within the dorsal HIP of adult rats stimulates neurogenesis and produces signs of stress resilience including enhanced extinction of conditioned fear. Conversely, viral vector-mediated elevation of SPRY2 expression intensifies the behavioral consequences of stress. Studies of these manipulations in HIP primary cultures indicate that SPRY2 negatively regulates fibroblast growth factor-2 (FGF2), which has been previously shown to produce antidepressant- and anxiolytic-like effects via actions in the HIP. Our findings strengthen the relationship between HIP plasticity and stress responsiveness, and identify a specific intracellular pathway that could be targeted to study and treat stress-related disorders.

## Introduction

Accumulating evidence suggests that the rate of neurogenesis in the hippocampus (HIP) plays an important role in the development, manifestation, and relief of depressive and anxiety disorders [1–3]. It is well established that stress, which can trigger psychiatric illnesses including major depression (MD) and post traumatic stress disorder (PTSD) in humans [4], decreases HIP neurogenesis in laboratory animals [5–6]. Conversely, various treatments with antidepressant effects (including selective serotonin reuptake inhibitors [SSRIs], norepinephrine reuptake inhibitors [NRIs], and electroconvulsive seizure [ECS]) can increase neurogenesis in the HIP of laboratory animals [2,3,5]. The efficacy of antidepressants is eliminated by ablation of HIP neurogenesis, suggesting that this process is critical for the relief of depressive signs [7,8]. The specific mechanisms by which HIP neurogenesis produces these effects are not yet clear, but may involve local changes in neuronal excitability [9] that promote processes such as cognitive flexibility [10]. Importantly, however, there is also evidence that standard antidepressants can produce therapeutic-like effects that are independent of neurogenesis [11–13]. Indeed, recent findings demonstrate that the relationship among stress, neurogenesis, and behavior is highly complex [14,15], and can depend on factors such as age [16], individual differences in coping mechanisms [17], and predictability of a stressor [18]. Thus while the available evidence suggests that neurogenesis can serve as a biomarker of neuroplasticity and stress resistance, additional research is needed to test this hypothesis in a way that provides deeper insight on the conditions under which it is—and is not—supported.

In humans, repeated administration of electroconvulsive therapy (ECT) is highly effective in the treatment of depressive disorders, producing therapeutic effects in up to 90% of patients [19]. In a previous study examining the effects of ECS (a laboratory model of ECT) on the prefrontal cortex (PFC), we found that repeated treatment increased glial cell numbers while concomitantly reducing expression of Sprouty2 (SPRY2) [20]. These findings raised the possibility that SPRY2 could be involved in the intracellular signaling processes that regulate key molecular and behavioral responses to ECS. SPRY2 is an intracellular factor that acts as a negative regulator of receptor-tyrosine-kinase (RTK)-dependent signaling pathways that are stimulated by neurotrophic factors implicated in cell proliferation, differentiation, and neurogenesis [21–23]. Growth factor binding at RTKs stimulates the extracellular signal-regulated/mitogen activated protein kinase (ERK/MAPK) cascade, an intracellular signaling pathway implicated in the effects of trophic factors including fibroblast growth factor (FGF) [24]. Stimulation of ERK/MAPK increases SPRY2 transcription and, in turn, activated (phosphorylated) SPRY2 exerts feedback inhibition of this pathway. A mutated form of SPRY2 in which tyrosine is replaced with phenylalanine at residue 55 (Y55F) has dominant-negative (dn) effects, enhancing ERK/MAPK activation and promoting processes such as differentiation and survival of immature neurons *in vitro* [21]. Although SPRY2 is an intracellular factor, the effects of altering its function may be related to an ability to regulate signaling pathways that control expression of proteins with extracellular actions. Little is known about the ability of SPRY2 to regulate these processes *in vivo* within adult brain.

In the present studies, we examined the effects of an ECS regimen that increases gliogenesis and decreases SPRY2 expression within the PFC [20] on neurogenesis and SPRY2 expression in the dorsal HIP. Upon finding dramatic alterations in both markers in the ECS studies, we then designed a separate, more comprehensive set of studies in which we used microinjections of a herpes simplex virus (HSV) vector to transiently elevate or disrupt SPRY2 function in this region. We selected HSV vectors specifically because they are neurogenic (preferentially expressed in neurons) and their effects are transient, peaking 3–4 days after transduction and becoming minimally detectable by 7 days post-transduction [25–29]. This approach enabled us to explore the long-term molecular and behavioral consequences of a brief pulse of altered SPRY2 function in neurons within the HIP. We now describe evidence that disruption of SPRY2 function in the dorsal HIP can produce antidepressant-like effects on molecular and behavioral metrics, and that these effects may be related to reduced inhibition of FGF2 expression.

## Methods

### Animals

Male Sprague-Dawley rats (250–300 g) were obtained from Charles River Laboratories (Wilmington, MA, USA) and housed in pairs on a 12/12-hr light/dark cycle with *ad libitum* access to food and water except during behavioral tests. All procedures were approved by the McLean Hospital Institutional Animal Care and Use Committee (IACUC) and conducted in accordance with the 1996 National Institutes of Health (NIH) Guide for the Care and Use of Laboratory Animals.

### Electroconvulsive seizure (ECS)

Rats were administered ECS as described previously [20]. Briefly, ECS-treated rats were given repeated seizures once daily for 10 days in the mornings by passing a 99-mA, 0.5-s, 100-Hz current via earclip electrodes, using a current generator (Ugo Basile, Comerio, Italy). Control (Sham)-treated rats received similar handling including attachment of ear clips but no current was passed. BrdU (Bromodeoxyuridine; Sigma, St. Louis, MO, USA) was administered intraperitoneally (IP) to mark cell proliferation at 50 mg/kg twice daily for the same 10-day duration as ECS administration. Following the last day of ECS and BrdU treatment, the rats were maintained for an additional 4 weeks with no interventions. Rats were then overdosed with pentobarbital (130 mg/kg, IP) and perfused transcardially with 4% paraformaldehyde, and brains were cut into 40-μm coronal sections (6 series per brain) on a sliding microtome. Immunohistochemistry was performed to quantify BrdU- and SPRY2-labeled cells as described [20]. During quantification, the investigator performing cell counts was not aware of the treatment conditions. Left and right HIP were quantified separately; the boundaries of this area were drawn on slides containing sections stained for BrdU or SPRY2-immunoreactive cells. Each series of HIP sections included 5–7 sections. Measurements were performed using a brightfield microscope (Zeiss Axioskop2, Oberkochen, Germany) and personal computer running StereoInvestigator software (MicroBrightField, Williston, VT, USA). HIP volume, cell density, and cell number were calculated as described [20].

### Viral Vectors

Herpes simplex virus (HSV) vectors encoding dn(Y55F)SPRY2, wildtype (wt)SPRY2, and ß-galactosidase (the protein product of the LacZ gene) were produced as described previously [27]. Each protein was tagged with an epitope (-flag) to facilitate detection in brain. The vectors were suspended in 10% sucrose, and the average titer of the purified viral stocks was 5.0 x 10^7^ infectious units (iu)/ml. These HSV vectors induce transgene expression that is maximal 2–3 days after gene transfer and waning by 6–7 days [27].

### Stereotaxic Surgery

Rats were anesthetized with pentobarbital (65 mg/kg, IP) and given subcutaneous atropine (0.25 mg/kg) to minimize bronchial secretions. For studies in which neurogenesis was quantified, rats received a unilateral microinjection (1.0 μl) of one of the HSV vectors into one hemisphere (randomly assigned) and a corresponding microinjection of HSV-LacZ into the other hemisphere. For the studies in which behavior was quantified, bilateral microinjections were used. The HSV vectors were delivered over 10 min into the dorsal HIP (flat-skull coordinates relative to bregma: AP = -3.6, ML = ±2.0, DV = -2.6 mm below dura). Some rats were used to examine the immediate effects of dnSPRY2 or wtSPRY2 expression (beginning 3 days after gene transfer), whereas others were used to examine the long-term effects of transient expression of these transgenes (beginning 28 days after gene transfer). For rats in the 28-day group, our working hypothesis was that any effects of the viral vectors would not be related to transduction of dividing cells, because HSV is neurotrophic and its effects would have waned long before any cells dividing at the time of transduction had differentiated into neurons, a process that normally requires ~3–4 weeks [30].

### Histological analysis of HSV vector effects

To label dividing cells, rats received a total of 6 injections of BrdU (100 mg/kg, IP) at 12-hr intervals beginning 7 days after surgery. Twenty-eight days after surgery, rats were overdosed with pentobarbital (130 mg/kg, IP) and transcardially perfused with 4% paraformaldehyde. Free-floating coronal sections (40 μm) containing dorsal HIP were incubated with mouse anti-BrdU antibody (1:1000; Chemicon, Temecula, CA, USA), biotinylated horse anti-mouse antibody (1:200; Vector Laboratories, Burlingame, CA, USA) and avidin-biotin (ABC Elite; Vector Laboratories). For fluorescent double-labeling, sections were incubated with rat anti-BrdU (1:100; Accurate Chemical, Westbury, NY, USA), mouse anti-NeuN (1:100; Chemicon), and rabbit anti-S100ß (1:500; Dako, Glostrup, Denmark) as described previously [20]. The fluorescent secondary antibodies used were (respectively) goat anti-rat Alexa Fluor 555 (1:200; Molecular Probes, Eugene, OR, USA), goat anti-mouse Alexa Fluor 488 (1:200; Molecular Probes), and goat anti-rabbit Alexa Fluor 633 (1:200; Molecular Probes). For quantification of BrdU labeling all BrdU positive cells in the DG were counted using a brightfield microscope and StereoInvestigator software. Only rats with microinjection placements localized within the dorsal HIP were used; cell counts were quantified in sections within which the infusion site was clearly visible. To determine net cell number, the number of BrdU-immunolabeled cells in the hemisphere treated with HSV-LacZ was subtracted from the number in the hemisphere that received HSV-dnSPRY2 or HSV-wtSPRY2. To confirm colocalization of BrdU and each cell marker, sections were analyzed on a confocal microscope (Leica TCS NT; Exton, PA, USA) as described previously [31]. A minimum of 20 BrdU-immunolabeled cells in the DG at the injection site were analyzed with Z-plane sectioning (1.5 mm steps).

### HIP Primary Cell Cultures and PCR

Adult rat hippocampal primary cultures were prepared as described previously [32]. Briefly, hippocampi from 6 adult rats were isolated and chopped into small pieces in HABG (Dissecting Media) with a sterile scalpel in order to aid in enzymatic dissociation of the cells. The tissue pieces were moved to a 15 ml polypropylene tube containing 2mg/ml papain dissolved in HABG. The tissue was then incubated at 30^o^ C in an incubator with a rotating apparatus, and triturated 10 times with a fire-polished sterile Pasteur pipette to release the cells. Larger chunks were allowed to settle, the supernatant was removed and the tissue chunks were re-suspended and triturated as above. Pooled supernatants of the cells were then separated in an OptiPrep density gradient (800 g for 15 min at room temperature). Fractions 2 and 3 were collected, washed and re-suspended in Neurobasal A/B27 growth media. Cells were counted and plated on poly-D-lysine coated glass coverslips. Plating density was 320 cells/mm^2^. Cells were allowed to settle for one hour and then washed twice with pre-warmed HABG. Coverslips were then transferred to wells containing pre-warmed growth media. One half of the volume of growth media was replaced every 4–5 days. Viral vector treatments were performed 7 days after plating.

For PCR, a Power SYBR Green Cells-to-CT Kit (Ambion, Austin, TX) was used to synthesize cDNA in a ThermoHybaid iCycler (Thermo Scientific) directly from total RNA within lysed hippocampal cell cultures. Quantitative real-time PCR (qRT-PCR) was performed using a BioRad MYiQ Real-Time PCR Detection System. DNA oligo primers (25nM standard, desalted) specific for genes encoding fibroblast growth factor 2 (Fgf2; Forward primer: GCGACCCACACGTCAAACTACAGC, Reverse primer: GAAGCCAGCAGCCGTCCATCTTC; product size, 211bp), brain derived neurotrophic factor (BDNF; Forward primer: AGCAGTCAAGTGCCTTTGGAGCC, Reverse primer: ATCTGCCGCTGTGACCCACTCG; product size, 168bp), tubulin, alpha 1A (Tuba1a; Forward primer: AGAAGCAACACCTCCTCCTCGC; Reverse primer: AGGCTGGATGCCATGTTCCAGG; product size, 154 bp), and 18S-RNA (Forward primer: TGGCTCAGCGTGTGCCTACC; Reverse primer: TAGTAGCGACGGGCGGTGTG; product size, 177bp) were used at a concentration of 3.0 μM. The primers were designed using NCBI Primer-BLAST (http://www.ncbi.nlm.nih.gov/tools/primer-blast/) and purchased from Integrated DNA Technologies (Coralville, Iowa). Melt curve analysis and polyacrylamide gel electrophoresis confirmed the specificity of the primers. PCR cycling conditions were 95°C for 10 min; 40 cycles at 95°C for 15 sec, 60°C for 1 min, 79°C for 15 sec. Data were collected at read temperatures of 83–88°C for 15 sec depending on amplicon melt temperatures. Standard dilution curves were generated for each primer set by serially diluting (1.00, 0.25, 0.0625, 0.0156, 0.0039-fold) a master cDNA stock comprising an equal mix of cDNA from all treatment groups. The log_10_ of the dilution values was plotted against the threshold cycle values to generate standard curves. MyiQ Optical System Software (BioRad) was used to analyze the data. Samples containing no cDNA template and samples from cDNA synthesis reactions that contained no reverse transcriptase (no RT) were run as controls for contamination and amplification of genomic DNA, respectively. Reported values were normalized to the average values of two internal controls: Tuba1a and 18S-RNA. Data are expressed as mean relative levels of the gene of interest/internal standards mRNA±SEM.

### Forced Swim Test (FST)

The FST studies were conducted as described previously [33] with minor modifications. The FST is a two-day procedure in which rats swim under conditions in which escape is not possible. On the first day, rats are placed in clear, 65-cm tall, 25-cm diameter cylinders filled to 48 cm with 25°C water for 15 min. Twenty-four hr later, rats are re-tested for 5 min under identical conditions. Swim tests were videotaped from the side of the cylinders and scored by raters unaware of treatment conditions. Each rat was rated at 5 sec intervals and the predominant behavior was assigned to one of 3 categories: immobility (making only movements necessary to keep the head above water), swimming (making swimming movements directed toward the center of the cylinder), or climbing (making forceful thrashing movements with the forelimbs directed against the walls of the cylinder).

### Open Field Locomotor Activity

One day after FST testing, locomotor activity was monitored in darkness for 1 hr in automated 43 x 43 x 31-cm (length x width x height) activity chambers (MED Associates, St. Albans, VT, USA) as described previously [34]. The main dependent variable was distance traveled (in cm) in 5-min bins during the test session.

### Fear-Potentiated Startle Test (FPS)

FPS was performed as described previously [34] with minor modifications. Habituation, training, testing, and re-testing (extinction) sessions were conducted in an automated FPS apparatus (Med Associates). During these sessions the rats were held in 19 x 9 x 14-cm Plexiglas cages with steel rod floor bars. Each cage was attached to a load-cell platform and was contained within a 64 x 40 x 60-cm ventilated sound-attenuating chamber. Rats were presented with startle stimuli (50 ms, 100 dB, 30 sec inter-trial interval) 4–6 days prior to surgery. These data were used to assign the rats to groups with similar baseline startle. On the training day (28 days post surgery) rats were placed in the apparatus and (following a 5-min acclimation period) presented with 10 light stimuli (conditioned stimulus [CS]) (3.7 sec) that co-terminated with a footshock (unconditioned stimulus [US]) (0.5 sec, 0.6 mA). At 2 and 4 days after training, rats were returned to the chambers and (after acclimation) presented with startle stimuli (60) in the presence or absence of the CS. The main dependent variable was fear potentiated startle (FPS), which was defined as the percent difference in startle magnitude elicited in the presence versus absence of the light.

### Elevated Plus Maze (EPM)

EPM was performed as described previously [34]. Rats were tested on a black plastic maze (Hamilton-Kinder, Poway, CA, USA) that was 110 x 110 x 85 cm (length x width x height). Each arm of the maze was 10 x 50 cm (width x length), and the intersection of the arms was 10 x 10 cm; the walls of the closed arms were 40 cm high. Each rat was placed in the center of the maze facing a closed arm and videotaped for 5 min. The sessions were videotaped and scored by raters unaware of treatment conditions. The main dependent measure was time (in sec) spent on the open arms.

### Morris Water Maze (MWM)

The MWM was conducted using a modification of previously published methods [35]. The water maze was a circular pool (180 cm diameter x 65 cm height) filled to 35 cm with water (25°C) made barely visible by non-toxic white paint. A clear plastic platform (10 cm diameter x 33 cm height) was placed in the north-east (NE) quadrant of the pool. For the acquisition test, rats received 2 training trials per day, with a different (semi-randomized) starting location (north, south, east, or west) each day. The maximum trial length was 90 sec; if the rat found the platform, it was left there for 30 sec, whereas if it did not, it was placed there for 30 sec. After 4 days of training, there was a probe test on day 5 during which the platform was removed and the rats were allowed to swim for 60 sec. There was then another 4-day training phase followed by a second probe test on day 10. Following the second probe test, the rats were tested for reversal learning: they received 4 training trials per day for 4 days, with the platform location changing each day. The sessions were videotaped and scored by raters unaware of treatment conditions. The main dependent measures were latency to find the platform during training trials, and time spent in the NE quadrant during probe tests.

### Statistical Analyses

To assess the possibility of an inverse relationship between cell proliferation and SPRY2 expression in the HIP, a repeated measures (cell type) linear regression analysis was performed using the log-transformed cell counts. The effects of the viral vector treatments on HIP cell phenotypes were analyzed using one-way analysis of variance (ANOVA) followed by *post hoc* Fisher’s protected *t*-tests (two-tailed). The effects of the vectors on FGF2 and BDNF mRNA levels were analyzed using separate one-way ANOVAs followed by *post hoc* Fisher’s protected *t*-tests (two-tailed). For the FST, group differences in the number of occurrences of each category of behavior were analyzed using one-way ANOVAs followed by *post hoc* Fisher’s protected *t*-tests (two-tailed). For the open field tests, group differences in distance traveled during the test session was quantified in 5-min increments and analyzed using a two-way (treatment X time) ANOVA with repeated measures (time). Percent FPS data were analyzed with both a one-way ANOVA (entire test session) and two-way (treatment X time) ANOVA with repeated measures (time) (10-min test blocks); significant effects and interactions were further analyzed using *post hoc* Fisher’s protected *t*-tests (two-tailed). For the EPM, percent time spent in the open arms was analyzed using a one-way ANOVA. For the MWM, group differences in mean latencies to find the platform over the 8 training days were analyzed using a two-way ANOVA with repeated measures, whereas group differences on the two probe tests were analyzed using a two-way ANOVA with repeated measures followed by Simple Main Effects tests. The alpha-level for all analyses was 0.05. Only rats in which guide cannulae were located in the DG were retained.

## Results and Discussion

**Fig 1:**
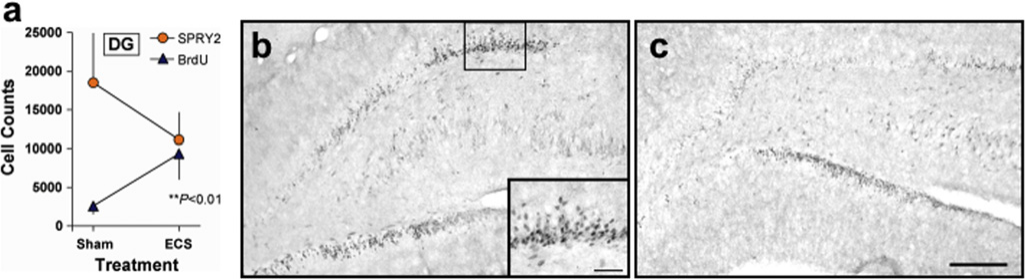
Effect of ECS on the dorsal HIP. (**a**) Repeated ECS increased the number of BrdU-labeled cells and decreased the number (mean ± SEM) of SPRY2-labeled cells within the DG (linear regression: *t*_8_ = 3.41, *P*<0.01); N = 5/group. (**b**) Expression of SPRY2 in the HIP of sham-treated rats at low or (*inset*) high magnification. (**c**) Expression of SPRY2 after repeated ECS. Scale bar: 200 μm or (*inset*) 50 μm.

**Fig 2:**
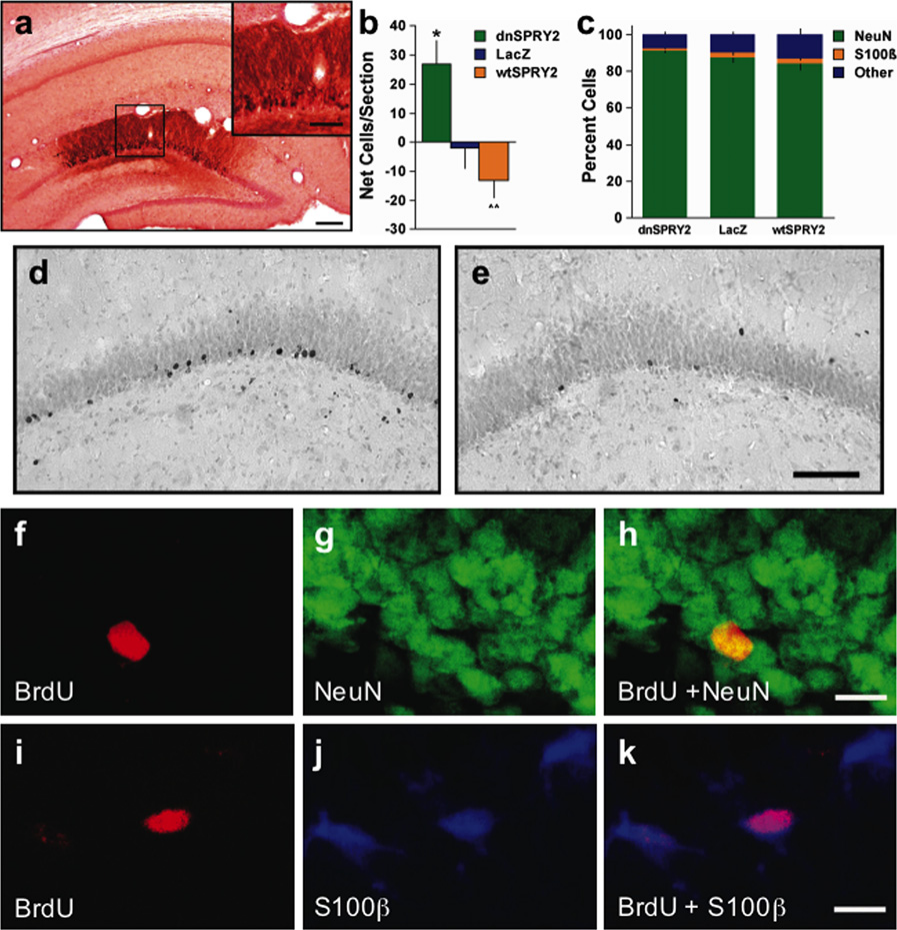
Effect of viral-mediated gene transfer in the dorsal HIP. (**a**) Detection of dnSPRY2 (tagged with a-flag epitope) 3 days after gene transfer (scale bars: 200 μm or [*inset*] 100 μm). (**b**) Net number (mean ± SEM) of BrdU-labeled cells (per 40 μm section) 28 days after gene transfer, expressed as the difference between the hemisphere treated with HSV-dnSPRY2, HSV-LacZ, or HSV-wtSPRY2 and the contralateral hemisphere treated with HSV-LacZ (*F*_2,28_ = 7.27, *P*<0.01). (**c**) Viral vector treatments did not differentially affect the percentage (mean ± SEM) of cells expressing neuronal (NeuN; *F*_2,28_ = 1.99, not significant), glial (S100ß; *F*_2,28_ = 0.98, not significant) or undefined (other; *F*_2,28_ = 1.57, not significant) cell markers. (**d**) Brightfield microscopy showing BrdU-labeled cells after HSV-dnSPRY2 or (**e**) HSV-LacZ. (**f**) Confocal microscopy showing a BrdU-labeled cell (red), (**g**) NeuN-labeled neurons (green), and (**h**) a double-labeled neuron (*overlay*, yellow; scale bar: 10 μm). (**i**) BrdU-labeled cells (red) were occasionally double-labeled with S100ß (**j**, blue) indicating glial cells (**k**, *overlay*, violet; scale bar: 10 μm). *P<0.05 compared to LacZ, ^^*P*<0.01 compared to dnSPRY2, Fisher’s protected *t*-tests, 9–11 rats/group.

Immunohistochemical analysis of brain sections from rats treated with the ECS regimen that affects gliosis and SPRY2 expression within the PFC [20] revealed that, as in the PFC, the number of BrdU-labeled cells increased as the number of SPRY2-labeled cells decreased within the DG of the dorsal HIP (Fig 1a–1c). This association led us to explore the possibility that reduced SPRY2 function within the HIP can cause cell proliferation and neurogenesis. We targeted an area of the DG within the dorsal HIP because focused X-ray irradiation of this area ablates neurogenesis and eliminates the antidepressant-like effects of fluoxetine [7]. Disruption of SPRY2 function within this area (Fig 2a) caused localized increases in cell proliferation that were evident 28 days after gene transfer, relative to a condition in which expression of *E*. *coli* ß-galactosidase was used to control for non-specific effects of gene transfer (Fig 2b, 2d and 2e). In contrast, viral vector-induced elevations of wildtype (wt)SPRY2 caused nominal (but not statistically significant) decreases in cell proliferation. The vast majority of the newly born (BrdU-labeled) cells expressed a neuronal marker (Fig 2c and 2f–2h), although a small number of double-labeled cells expressed a glial marker (Fig 2c and 2i–2k) [5,7]. The relative proportions of these and other (unidentified) cell types were not differentially affected by altered SPRY2 function (Fig 2c), suggesting that normal patterns of differentiation are preserved despite general elevations in proliferation rates. This pattern is consistent with that previously reported for neurogenesis-inducing regimens of standard antidepressant drugs, which also do not alter the relative proportions of these cell types [7]. These findings establish that transient disruption of SPRY2 function within the dorsal HIP results in increased neurogenesis.

**Fig 3:**
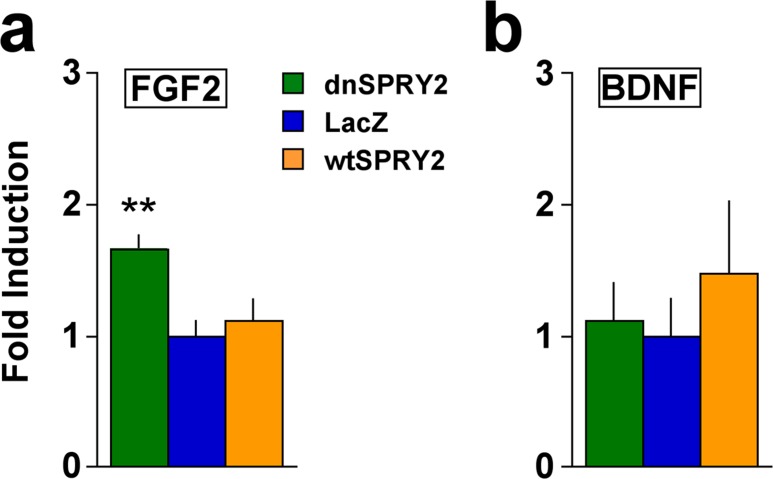
Expression of growth factor mRNA after treatment of HIP primary cell cultures with viral vectors. Data are presented as fold induction of the gene of interest (normalized to mean starting quantity of reference genes) relative to LacZ controls, ±SEM. (**a**) Viral vector treatment altered expression of FGF2 mRNA (*F*_2,22_ = 6.11, *P*<0.01); levels were significantly higher after dnSPRY treatment. (**b**) The same vectors had no effect on BDNF mRNA (*F*_2,15_ = 0.33, not significant). **P<0.01 compared to LacZ, Fisher’s protected *t*-tests, N = 8–9 per condition.

To confirm that disruption of SPRY2 function can regulate expression of growth factors as previously described [24], we conducted *in vitro* studies in HIP primary cultures. Expression of dnSpry2 increased mRNA levels of FGF2, which has been shown to produce antidepressant- and anxiolytic-like effects via actions in the HIP [36,37] (Fig 3a). Surprisingly, there was no effect of wtSPRY2 on FGF2 levels, raising the possibility that our model system was best suited to detect increases in FGF2 expression. None of the vectors affected mRNA levels of BDNF (Fig 3b), indicating that the effects of dnSPRY2 does not reflect a non-specific enhancement of growth factor gene expression.

**Fig 4:**
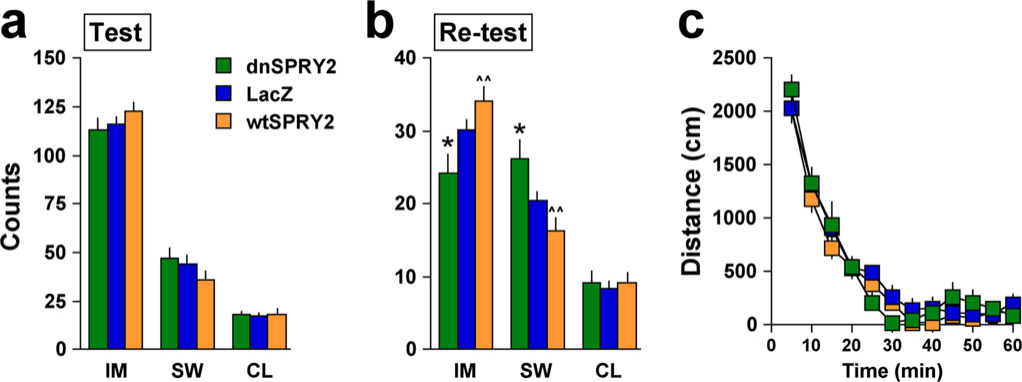
Forced swimming and locomotor activity behaviors 28 days after gene transfer. (**a**) During the first exposure to the FST, there were no differences in immobility (IM; *F*_2,41_ = 1.20, not significant), swimming (SW; *F*_2,41_ = 1.60, not significant) or climbing (CL; *F*_2,41_ = 0.05, not significant) behaviors (mean ± SEM) among rats given the dnSPRY2, LacZ, or wtSPRY2 vector into the dorsal HIP. (**b**) Differences in immobility (*F*_2,41_ = 5.77, *P*<0.01) and swimming (*F*_2,41_ = 6.18, *P*<0.01) emerged upon re-exposure to forced swimming 24 hr later. (**c**) Vectors did not affect activity in an open field (*F*_22,451_ = 0.09, not significant). *P<0.05 compared to LacZ, ^^*P*<0.01 compared to dnSPRY2, Fisher’s protected *t*-tests, 14–15 rats/group.

Ablation studies provide evidence that HIP neurogenesis is necessary for the therapeutic effects of standard antidepressants [7]. However, direct evidence that neurogenesis in this region is sufficient to produce antidepressant (or otherwise beneficial) effects is lacking, in part because treatments that promote this process (e.g., ECS, SSRIs, NRIs) cause a variety of neuroadaptations throughout the brain. We used microinjections of the dnSPRY2 vector to determine if a treatment that increases neurogenesis restricted to the dorsal HIP produces antidepressant-like effects in rats using the forced swim test (FST), a stress-based behavioral model that identifies treatments with antidepressant effects in humans [38]. When behavioral testing was conducted 28 days after gene transfer—a time point by which cell proliferation triggered by the treatment can be differentiated into neurons [2]—there were no group differences in behavior during the first exposure to forced swimming (Fig 4a), indicating similar sensitivity to the acute effects of this stressor. Upon re-testing the following day, however, rats treated with dnSPRY2 showed reduced immobility and more swimming behavior (Fig 4b), an effect indistinguishable from that caused by standard antidepressant drugs [33]. Treatment with wtSPRY caused nominal (non-significant) increases in immobility during re-exposure to swim stress. Open field tests indicate no differential effects of the viral vectors on locomotor activity (Fig 4c), ruling out the possibility that alterations in SPRY2 function produce non-specific activating effects that complicate interpretation of FST data. When testing was instead conducted 3 days after gene transfer—a time not sufficient to allow differentiation of treatment-induced cell proliferation to differentiate into neurons [2]—there were no effects in the FST or open field (not shown), confirming that alterations in cell proliferation or SPRY2 function alone are not sufficient to produce changes in these behaviors. These findings establish that disruption of SPRY2 function is sufficient to produce antidepressant-like effects and reduce the expression of behavioral alterations triggered by stress, thereby strengthening associations between this process and the therapeutic effects of treatments commonly used to treat clinical depression.

**Fig 5:**
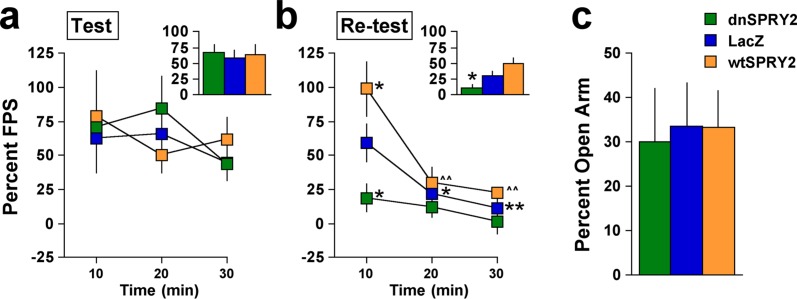
Fear and anxiety-like behaviors 28 days after gene transfer. (**a**) During the first test session, there were no differences in the time course (*F*_4,40_ = 0.71, not significant) or overall mean (*inset*: *F*_2,20_ = 0.13, not significant) of FPS (mean ± SEM). (**b**) Upon re-testing 48 hr later, there were differential effects of the viral vectors in the time course (*F*_4,40_ = 2.63, *P*<0.05) and overall mean (*inset*: *F*_2,20_ = 7.61, *P*<0.01) of FPS. (**c**) In separate rats, there were no differences in time spent on the open arms of the EPM (*F*_2,20_ = 0.34, not significant), *P<0.05, **P<0.01 compared to LacZ at the 10-min time point, ^^*P*<0.01 compared to wtSPRY2 at the 10-min time point, Fisher’s protected *t*-tests, 7–8 rats/group.

Because depression is often co-morbid with anxiety in humans, we also examined the effects of altering SPRY2 function in the HIP on fear-potentiated startle (FPS), a model used to study stress-induced fear and anxiety-related states [34,39]. In this model, rats learn during training sessions that a previously neutral stimulus (a light) predicts footshock. Fear is subsequently reflected in test sessions by an enhanced startle response to white noise bursts in the presence of the light, which is no longer paired with the footshock. When rats were trained with 10 light-footshock pairings 28 days after gene transfer and tested 48 hr later, there were no differential effects on FPS (Fig 5a). These data suggest that our methods of influencing the rate of neurogenesis in the dorsal HIP have no effect on encoding, consolidation, or retrieval of stress-induced memories, although chemical or radiological methods that arrest neurogenesis within this region can disrupt fear conditioning [40]. Upon re-testing 48 hr later, however, rats treated with dnSPRY2 showed reduced FPS (Fig 5b, inset), raising the possibility that increased neurogenesis in the dorsal HIP facilitated the extinction of fear [39,41], a form of new learning that occurs in response to the repeated presentations of the light in the absence of footshock. Detailed time course analysis of the re-test session (Fig 5b) also revealed a period of elevated fear in rats treated with wtSPRY2, suggesting that even small reductions in the rate of neurogenesis in the dorsal HIP can disrupt extinction and render rats more vulnerable to the long-term behavioral effects of stressors. Interestingly, these treatments did not differentially affect anxiety-related behavior when rats were instead tested in the elevated plus maze (EPM) (Fig 5c), a model that identifies in rats treatments with anxiolytic effects in humans [34].

**Fig 6:**
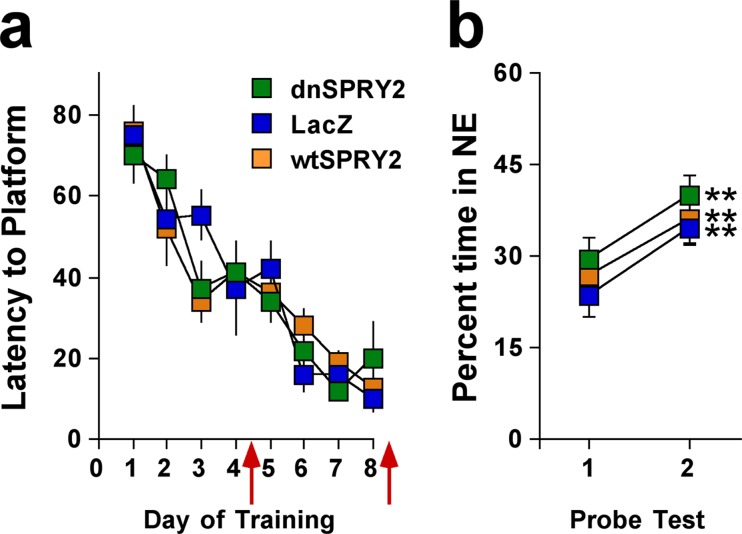
Learning and memory behaviors beginning 28 days after gene transfer. (**a**) Over the course of training in the MWM, there we no group differences in latencies to find the platform (main effect of treatment: *F*_2,27_ = 0.99, not significant; interaction: *F*_14,189_ = 1.54, not significant), and latencies decreased over days (*F*_7,189_ = 40.2, *P*<0.01). (**b**) There were no group differences in the time spent in the quadrant within which the platform was located (Northeast; NE) in probe tests conducted after training sessions 4 and 8 (red arrows) (main effect of treatment: *F*_2,27_ = 1.08, not significant; interaction: *F*_2,27_ = 0.23, not significant), and times increased between probe tests 1 and 2 (main effect of trial: *F*_1,27_ = 40.3, *P*<0.01). Times were higher during the second probe test for rats given the dnSPRY2 (*F*_1,10_ = 13.1, *P*<0.01), LacZ (*F*_1,10_ = 29.1, *P*<0.01), and wtSPRY2 (*F*_1,10_ = 12.3, *P*<0.01) vector. **P<0.01 within-subject comparison with probe trial 1, Simple Main Effects tests.

**Fig 7:**
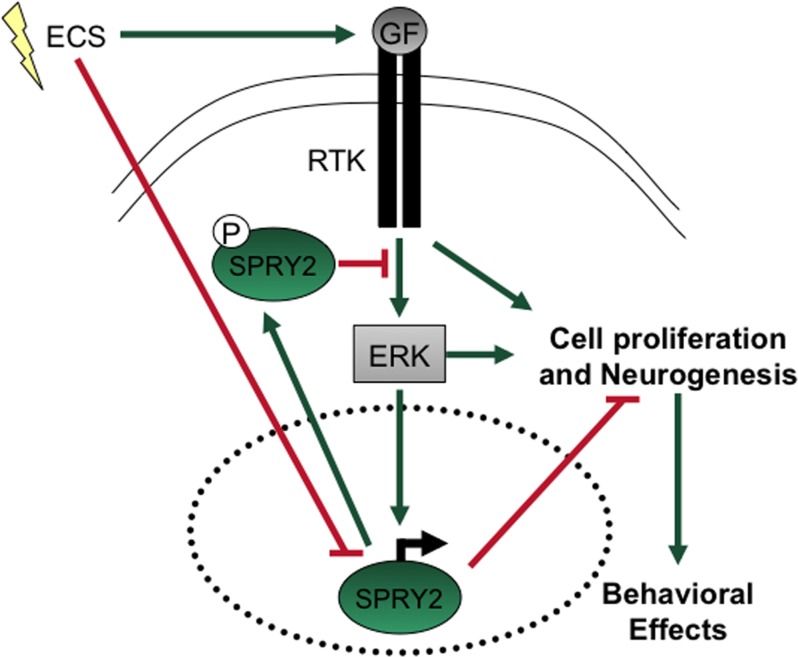
Highly simplified schematic depicting a potential pathway by which disruption of SPRY2 function produces elevated neurogenesis and antidepressant-like behaviors. Binding of growth factors (GF) such as FGF2 at RTKs activates ERK-dependent signaling cascades (green arrows), resulting in processes that tend to increase neurogenesis and produce antidepressant-like behaviors. However, this process also results in elevated SPRY2 transcription and activation (phosphorylation), which tends to inhibit these processes. Electroconvulsive seizure (ECS) activates GFs and inhibits SPRY2, both of which promote neurogenesis and antidepressant-like behaviors.

To determine if the effects of altered SPRY2 function on FPS represent more general effects on learning and memory, we tested rats in the MWM, a task that involves spatial learning [35,42]. There were no group differences in the rate at which the rats acquired that task (Fig 6a). In addition, rats in all groups spent more time within the quadrant in which the platform was normally located during the second probe test than in the first, a broad indication of normal learning (Fig 6b). When these same rats were subsequently re-trained in reversal tests to find the platform in a new location, there were also no group differences (not shown). Together, these findings suggest that disruption of SPRY2 in the dorsal HIP specifically affect brain processes involved in new learning of fear inhibition—without having general effects on anxiety, learning, or memory [39,41]—thereby enhancing resilience and facilitating recovery from stress. When our molecular and behavior findings are considered in the context of previous work, it is possible to design a model that can be used to test working hypotheses in future studies (Fig 7).

## Conclusions

We show that ECS—which models key aspects of ECT, a treatment with particularly high efficacy in people with depressive disorders [19]—produces a downregulation of the growth factor inhibitor SPRY2 in the dorsal HIP. We also show that a prominent consequence of the ability of ECS to downregulate SPRY2 function—as modeled by expression of a dominant negative form of SPRY2—is elevated neurogenesis in the dorsal HIP and behavioral effects that may reflect resistance to stress (i.e., the center and right side of Fig 7). These findings suggest that inhibition of SPRY2 function removes a cellular “brake” and causes a shift toward enabling neurogenesis and behavioral effects that resemble those seen after chronic treatment with antidepressants. The mechanisms by which ECS produces downregulation in SPRY2 (i.e., the left side of Fig 7) is not understood, and represents a separate question that requires additional research.

Our studies represent an early but important step in understanding of how the actions of SPRY2 within the HIP can affect behavioral responses to stress. As is often the case with new lines of work, there are important interpretational limitations that can be addressed in future work. As one example, we used HSV with the specific intention of studying the consequences of a brief (3–4 day) “pulse” of altered SPRY2 within the HIP. Our prediction was that changes in SPRY2 signaling in mature cells might affect expression of growth factors such as FGF2, which has extracellular actions that are involved in the same types of neural processes that have been described for SPRY2 [36,37]. While it is possible that some of our findings could reflect the effects of the HSV vectors on dividing cells, we feel that this is unlikely because any dividing cells infected by the HSV vectors were at least several weeks from differentiating into neurons [30]. The fact that expression of genes encoded by these HSV vectors is both highly transient and neurogenic [25–29] mitigates concerns that our findings are due to effects on dividing cells. Replicating and extending these studies with other viral vectors that enable more persistent transgene expression may provide additional insight on our findings. Indeed, use of long-acting retroviral vectors may enable cell labeling that helps to determine whether our effects are mediated via effects on dividing or non-dividing cells [43] or changes within highly specific subregions of the HIP [44]. In addition, recent studies involving such vectors have shown that increases in HIP neurogenesis are functional and can produce effects on fear-related behaviors that are broadly consistent with those seen in our studies by causing increases in synaptic contacts [45], highlighting the utility of this complementary approach for hypothesis testing. As another example, SPRY2 has multiple phosphorylation sites that can affect its function [46]. While our *in vivo* findings with the Y55F dominant negative mutation are perfectly consistent with *in vitro* findings seen with the same construct, the use of other mutations to affect SPRY2 function will enable more comprehensive hypothesis testing. Such studies are beyond the scope of the present research, however, which was designed primarily to follow up on our initial finding the ECS downregulates SPRY2 function in an area of the brain strongly associated with neurogenesis.

The present work is important because it may provide new insight on the neurobiology of stress-related disorders and their treatment. There is substantial evidence in humans that depressive and anxiety-related disorders are associated with aberrant HIP size or volume [1]. Brain imaging studies suggest HIP atrophy is associated with major depression or exposure to stressors including childhood maltreatment and warfare [47–49]. Moreover, smaller HIP volume predicts vulnerability to the pathological effects of stress [50]. Although it is not clear if these anatomic differences are associated with altered neurogenesis—due to difficulties in identifying newly-born cells in living humans—they may contribute to deficits in context-dependent learning processes such as extinction [51,52]. Behavioral interventions in mice that utilize safety cues to extinguish fear are associated with increased HIP neurogenesis [41]. If similar mechanisms apply in humans, directly or indirectly stimulating neurogenesis might help treat syndromes (e.g., PTSD) typified by persistent and debilitating conditioned fear or anxiety [39,51,52]. It is important to emphasize that the mechanisms by which ECS reduces SPRY2 function are not understood; indeed, this work provides a basis for future studies that address this gap in knowledge. Likewise, not all treatments that stimulate neurogenesis necessarily share common molecular mechanisms [53]; indeed, we found that a regimen of chronic desipramine (an NRI) sufficient to stimulate cell proliferation and neurogenesis did not alter SPRY2 expression (not shown). Differences in the molecular effects of ECT and standard antidepressants likely contribute to differences in their clinical efficacy [19]. In addition, evidence that antidepressants can produce therapeutic-like effects that are neurogenesis-independent in animal models [12] raises the possibility that it is not the birth of new neurons that is critical, per se, but rather changes to the local milieu that creates conditions conducive to more subtle cellular processes (e.g., enhanced synaptic connections in existing neurons). Regardless, control over the milieu that regulates cell proliferation and neurogenesis in adult brain may enable protection or repair of neuronal circuits often damaged by stress, trauma, or disease.
